# D614G substitution at the hinge region enhances the stability of trimeric SARS-CoV-2 spike protein

**DOI:** 10.6026/97320630017439

**Published:** 2021-03-31

**Authors:** Arangasamy Yazhini, Das Swayam Prakash Sidhanta, Narayanaswamy Srinivasan

**Affiliations:** 1 Molecular Biophysics Unit; Indian Institute of Science; Bangalore, Karnataka, 560012, India

**Keywords:** SARS-CoV-2, COVID-19, spike protein, D614G variant, mutation, normal mode analysis, residue contacts, frustration index, protein stability

## Abstract

Mutations in the spike protein of SARS-CoV-2 are the major causes for the modulation of ongoing COVID-19 infection. Currently, the D614G substitution in the spike protein has become dominant worldwide. It is associated with higher infectivity than the ancestral (D614)variant. We demonstrate using Gaussian network model-based normal mode analysis that the D614G substitution occurs at the hinge region that facilitates domain-domain motions between receptor binding domain and S2 region of the spike protein. Computer-aided mutagenesis and inter-residue energy calculations reveal that contacts involving D614 are energetically frustrated. However, contacts involving G614 are energetically favourable, implying the substitution strengthens residue contacts that are formed within as well as between protomers. We also find that the free energy difference (ΔΔG) between two variants is -2.6 kcal/mol for closed and -2.0 kcal/mol for 1-RBD up conformation. Thus, the thermodynamic stability has increased upon D614G substitution. Whereas the reverse mutation in spike protein structures having G614 substitution has resulted in the free energy differences of 6.6 kcal/mol and 6.3 kcal/mol for closed and 1-RBD up conformations, respectively, indicating that the overall thermodynamic stability has decreased. These results suggest that the D614G substitution modulates the flexibility of spike protein and confers enhanced thermodynamic stability irrespective of conformational states. This data concurs with the known information demonstrating increased availability of the functional form of spikeprotein trimer upon D614G substitution.

## Background

COVID-19 pandemic, the causing agent severe acute respiratory syndrome coronavirus-2 (SARS-CoV-2) virus harbours mutations and is linked to geographical-specific etiological effects [1,2].Although vaccination has begun in many parts of the world, the emergence of more infectious new variants of SARS-CoV-2 from different geographical locations such as B.1.1.7 from England, B.1.351 from South Africa and P.1 from Brazil continues to challenge our combat against COVID-19 [3]. These variants harbour several mutations in the genome and some of them present at the spike protein of the virus. Spike protein of SARS-CoV-2 is a 1273aa long transmembrane glycoprotein and comprises of three modules namely a large ectodomain that protrudes from the surface, a single-pass transmembrane anchor and a short intracellular tail.The ectodomain has S1 and S2 regions responsible for host cell binding and viral-host membrane fusion, respectively. At the junction of S1 and S2 regions, S1/S2 cleavage site is present and within the S2 region, S2' cleavage site is located. S1 region comprises of N-terminal domain (NTD, 27-305), receptor binding domain (RBD, 331-528), C-terminal domain 2 (CTD2, 529-590) and C-terminal domain 3 (CTD3, 591-685). Depending on RBD orientation in the S1 region, protomers in the functional form of spike protein trimer adopt closed or open conformation [4].Upon open conformation, RBD exposes angiotensin-converting enzyme 2 (ACE2) receptor binding region and interacts with a peptidase domain of the ACE2 receptor. This primary step clasps the virus onto the host surface [4]. Subsequent proteolysis at the S1/S2 cleavage site sheds the S1 region from the spike protein and cleavage at the S2' site near fusion peptide causes a large conformational change in the S2 region. Such conformational change leads to the insertion of fusion peptide to the host membrane and a six-helix bundle formation. At this state, spike protein bridges the viral envelope and host membrane. Hairpin-like bend in the S2 region brings both membranes to close proximity for fusion, following which genetic material gets injected into the cytoplasm of the host cell [5]. It is also noted that due to the multibasic nature of the S1/S2 cleavage site, the SARS-CoV-2 spike protein can be preactivated by the furin enzyme during viral packaging [6]. In contrast to SARS-CoV infection, this process reduces the dependence of SARS-CoV-2 on target cell proteases for the succeeding infection.Therefore, mutations in the spike protein that influence the initial steps for viral infection are associated with altered virus transmissibility and pathogenicity [1,7]. Early in the pandemic, the emergence of a new variant having D614G substitution in the spike protein was identified in China. Subsequently, the ancestral variant with aspartate at 614th position (SD614) has been asynchronously superseded by the glycine variant (SG614) worldwide [8]. This substitution is retained in the newly emerged variants (B.1.1.7,B.1.351 and P.1), indicating its positive selection [9]. Several studies in the past few months demonstrate that the D614G substitution enhances the infectivity and efficient replication of the virus [8,10-12]. The substitution disrupts a salt bridge interaction between aspartate at the 614th position and threonine at the 859th position of an adjacent protomer, thereby alters local inter-residue interactions [12]. It prevents premature shedding of the S1 region while promotes protease cleavage at the S1/S2 site and increases the conformational sampling of open conformation [13,14]. Therefore, it is of interest to document the effect of D614G substitution on the structural flexibility, inter-residue interaction energies and thermodynamic stability of the spike protein trimer.

## Methodology

### Gaussian network model-based normal mode analysis:

To identify regions that precisely act as hinges for domain-domain motions facilitating the transition between closed and open conformations, we performed Gaussian network model (GNM)-based normal mode analysis (NMA). GNM-NMA is a robust method to accurately predict hinges [15] and the calculations were performed for closed (protein data bank or PDB code: 6VXX) and 1-RBD up conformations (PDB code: 6VYB). The slowest normal mode was considered for analyzing Gaussian dynamics of the spike protein trimer.

### 3-D structural model for D614G variant of the spike protein trimer:

We generated an in silico model for the D614G variant of spike protein trimer using structure editing tool in UCSF chimera with default parameters [16]. Sidechains were optimized using SCWRL 4.0 program [17]. Two D614G variant models were generated corresponding to closed and 1-RBD up conformations of the spike protein trimer based on the reference cryo-EM structures available in the PDB entries 6VXX and 6VYB, respectively. Besides, spike protein structures with D614G substitution have been released at the time of our study [14] and hence were included in this analysis. Calculation of frustration index in the local residue contacts:The effect of D614G substitution on local interaction energies was examined using Frustratometer algorithm [18], which follows the principle that a native protein comprises several conflicting residue contacts resulting in local frustration. Based on Cβ distance, the residue contacts are categorized as short range (<6.5Å), long range (6.5-9.5Å) and water-mediated (long range and exposed to solvent).The algorithm computes the frustration index for a given residue or residue contact. The value below -1 indicates that the interacting pair is highly frustrated, while the index between -1 to 0.78 or above 0.78 indicates that the interacting pair is neutrally or minimally frustrated, respectively. The frustration index is calculated at three levels namely 'mutational', 'configurational' and 'single-residue'. In the mutational frustration, other residue types replace residue type, while in the configurational frustration, all possible interaction types between the native residue pairs were sampled through altering residue configuration. In the case of single-residue level, only other residue types for frustration index calculation replace a single residue site. Here, we analyzed all categories of frustration indices for the ancestral and dominant variants of spike protein trimer (SD614 and SG614) in closed and 1-RBD up conformations.

### Calculation of thermodynamic stability of SD614 and SG614 variants:

To study the effect of D614G variation on the thermodynamic stability of the spike protein trimer, we calculated free energy changes upon aspartate to glycine substitution using buildmodel function in FoldX [19]. Five iterations of free energy calculations were carried out to obtain converged results. The free energy changes were also calculated for the reverse scenario where glycine was substituted to aspartate in cryo-EM structures of SG614 (PDB codes: 7KDK and 7KDL). Inferences of the results were derived from closed and 1-RBD up conformations of the spike protein trimer.

## Results and Discussion:

**Figure 1:**
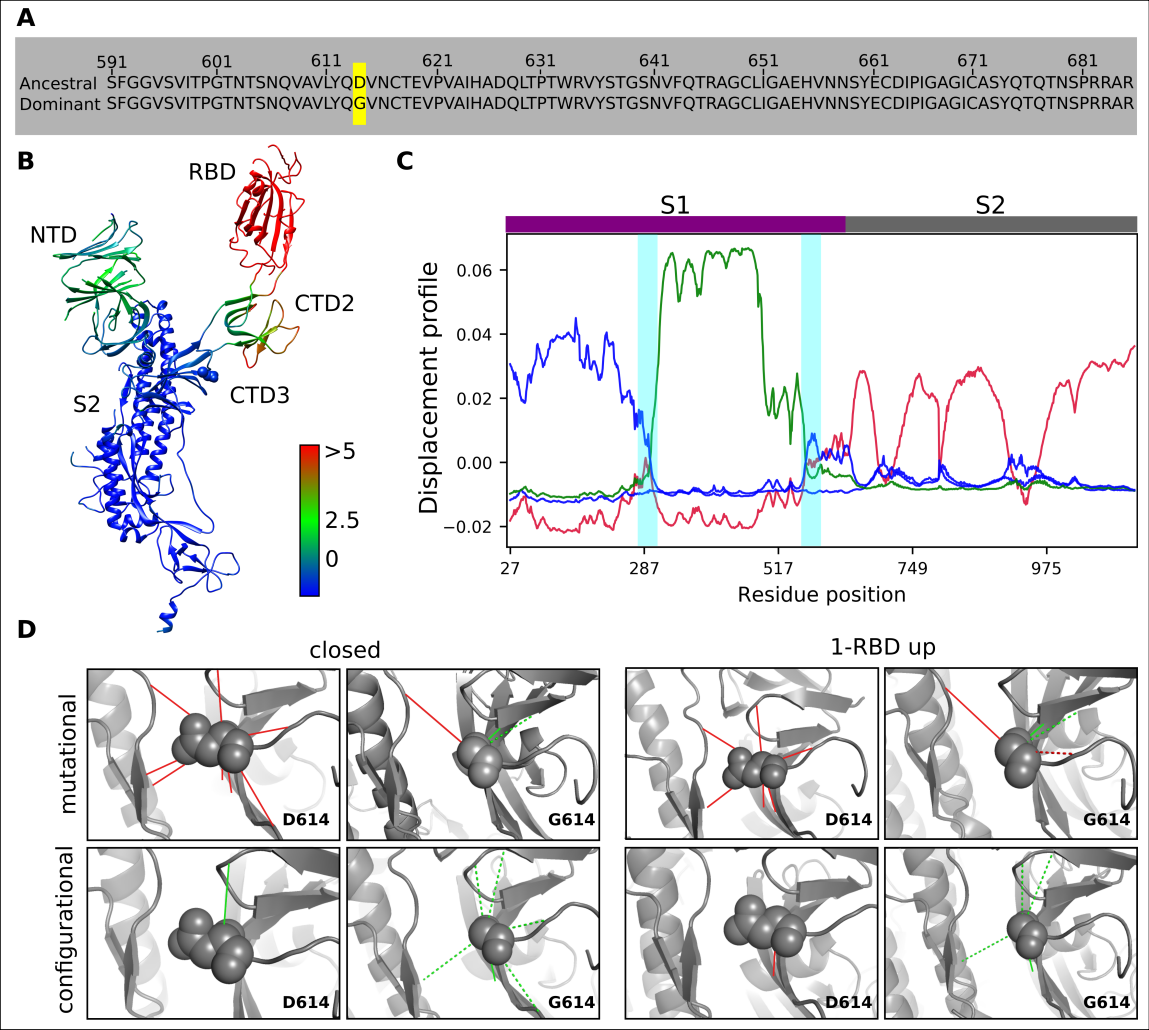
The significance of D614G substitution on the protein flexibility and interaction energy of residue contacts. A) Sequence alignment of CTD3 domain (591-685) between the ancestral (D614) and glycine (G614) variants of the spike protein. A yellow background highlights the substituted site. B) Cartoon representation of spike protein in RBD up conformation with domains labelled and colored based on the Cα deviation with respect to closed conformation. Color scale blue to red indicates Cα deviation (in Å) from low to high. The position 614 is highlighted by the sphere representation of the residue. C) Shown as a line plot is the displacement profile of protomers in the closed (crimson) and 1-RBD up (green- open; blue- closed) conformations. Hinges in the S1 region are highlighted with vertical cyan bars. S1 (magenta) and S2 (grey) regions are indicated by horizontal bars at the top of the plot. D) Shown is the mutational (top panel) and configurational (bottom panel) frustrations that exist in the inter-residue contacts formed by aspartate or glycine at position 614. Green and red lines indicate minimally and highly frustrated interactions, respectively. Water-mediated interactions are represented as dashed lines and the variant residue is shown as a sphere. Results from closed and 1-RBD up conformations were shown only for a protomer (chain ID: A) within the trimer since similar patterns are observed for the other two protomers (Supplementary Table S1).

The D614G substitution is present in the CTD3 domain and is highlighted in the sequence alignment between ancestral and dominant variants (Figure 1A). Comparison of spike protein in closed and 1-RBD up conformations shows that besides RBD undergoing a large displacement between these two states, the NTD and CTD2 domains have considerable Cα deviations with RMSD values 1.6Å and 3.4Å, respectively (Figure 1B). However, CTD3 superposes well (RMSD 0.6Å) and hence likely facilitates domain-domain motions.To understand the association of position 614 to protein flexibility,we analyzed displacement profiles of the slowest Gaussian mode of spike protein trimer derived from GNM-NMA. The crossover of displacement profiles from negative to positive or vice versa signifies the presence of a hinge. We observe that the crossover occurs consistently around Lys310-Phe318 (hinge-1) and Gly593-Val618 (hinge-2) regions in the CTD3 of both conformations and hence they serve as hinges (Figure 1C). The hinge-1 mediates NTDRBD motions while the hinge-2 mediates RBD-S2 motions (Figure 1C). Given that glycine is a highly flexible residue, the substitution potentially influences the flexibility of hinge-2, thereby modulates RBD-S2 motions essential for closed to open transition. We analyzed the effect of D614G substitution on the interaction energies by computing the frustration index of residues in the SD614 and SG614. As shown in Table 1, the frustration index of aspartate at 614th position in the SD614 is less than -1 for three protomers in closed and 1-RBD up conformations. Hence, in both the states,aspartate is highly frustrated. Conversely, the glycine in SG614 is neutrally frustrated, with the index values ranging from -1 to 0.78.This result was affirmed by similar observations when we analyze the cryo-EM structures of SG614 (released at the time of our analysis) [14]. It means that the frustrated residue at position 614 has become neutral upon the glycine substitution.

contacts. In both conformations of SD614, aspartate forms several intra- and inter-protomer contacts through direct, long-range electrostatic or water-mediated interactions (Table S1A), of which 8 are highly frustrated (Figure 1D, top panel). In the case of SG614 in closed conformation, the glycine has two minimally frustrated contacts with Leu611 and Cys649 of the same protomer and a highly frustrated contact with Pro862 of an adjacent protomer (Figure 1D, top left panel). In the 1-RBD up conformation of SG614, the glycine has the same contacts, as observed in the closed conformation, in addition to another contact with Asn616. The contacts with Leu611 and Cys649 are minimally frustrated (Figure 1D,top right panel). Similar observations are seen for the cryo-EM structures of SG614 (Table S1A). Therefore, the overall number of highly frustrated contacts is reduced upon aspartate to glycine substitution.

The configurational frustration index, indicating how favourable a native contact relative to other possible contacts between interacting residue pairs, further shows that in the closed conformation, aspartate (SD614) has one minimally frustrated contact with Arg646 (Figure 1D, left bottom panel). In contrast, glycine (SG614)has six minimally frustrated contacts (Fig. 1D, left bottom panel).Similarly, aspartate in 1-RBD up conformation of SD614 has a highly frustrated contact with Gly593, while upon glycine substitution (SG614), this contact becomes minimally frustrated. In addition, this residue forms three other minimally frustrated contacts with Thr645, Arg646 and Thr859 ( 1D, right bottom panel). Moreover, configurational indices calculated from the cryo-EM structures of SG614 indicate that glycine has three minimally frustrated contacts in both conformations and corroborate with the results from in silico models (Table S1B). Hence, glycine has more favourable contacts than aspartate between S1 and S2 regions of the same and adjacent protomers. Overall, our calculations of single residue, mutational and configurational frustrations reveal that glycine substitution modifies the local interaction energy in a favourable direction. Further, our calculations of the free energy changes upon D614G substitution show that the total free energy differences (ΔΔG) between two variants of spike protein trimer are - 2.6 kcal/mol and -2.0 kcal/mol for closed and 1-RBD up conformations, respectively. The differences are higher than the reasonable threshold ±0.5 kcal/mol and hence the substitution stabilizes the spike protein [20]. When we perform reverse mutation in SG614 structures, the differences have positive values i.e.,6.6 kcal/mol and 6.3 kcal/mol for closed and 1-RBD up conformations respectively, implying destabilizing effect. Together,it suggests that the glycine substitution creates a favourable local environment and enhances the overall stability of the spike protein trimer. Such effect may confer the increased availability of a functional form of spike protein, resulting in higher infectivity than the SD614 as reported by recent experimental studies [8,10,13].

## Conclusion

The D614G substitution in the SARS-CoV-2 spike protein, which is being intensively studied across the globe for COVID-19 prophylaxis and treatment, is under positive selection in the ongoing pandemic. This computational study demonstrates that D614G substitution occurs at the hinge region and potentially influences the conformational transition essential for human ACE2 receptor binding. Glycine changes the energetically frustrated local environment into favourable conditions for contacts present between S1 and S2 regions of the same protomer as well as from adjacent protomers. Consequently, the free energy of SG614 is lower than that of SD614, indicating that the local changes in the interaction energies at the 614th position in each protomer have a significant effect on the thermodynamic stability of the spike protein trimer.Our computational work based on a theoretical framework provides for the first time the protein flexibility and residue interaction energy-based rationale for the enhanced stability of the spike protein. These new findings add to the existing knowledge on the mechanism of increased transmissibility of SG614 and would help further investigations on the influence of additional substitutions acquired in the newly emerged SARS-CoV-2 variants of concern.

## Figures and Tables

**Table 1:** Single-residue level frustration index of aspartate and glycine in the SD614 and SG614, respectively. Results are shown for closed and 1-RBD up conformations.

Frustration index of the residue at 614th position	SG614		SG614 (in silico models)		SG614 (Cryo-EM structures)	
Conformational state	Closed	1-RBD up	Closed	1-RBD up	Closed	1-RBD up
Protomer 1	-1.25	-1.24	-0.48	-0.5	-0.55	-0.75
Protomer 2	-1.25	-1.31	-0.42	-0.35	-0.19	-0.39
Protomer 3	-1.3	-1.28	-0.46	-0.37	-0.88	-0.31
